# Editorial: Interdisciplinarity in internal medicine from basic investigations to molecular analysis

**DOI:** 10.3389/fimmu.2026.1883625

**Published:** 2026-06-16

**Authors:** Vlad Pădureanu, Anca Bobîrcă, Ştefan Lucian Popa

**Affiliations:** 1 Department of Internal Medicine, University of Medicine and Pharmacy of Craiova, Craiova, Romania; 2 Department of Internal Medicine and Rheumatology, ‘Carol Davila’ University of Medicine and Pharmacy, Bucharest, Romania; 3 2nd Medical Department, “Iuliu Hatieganu” University of Medicine and Pharmacy, Cluj-Napoca, Romania

**Keywords:** inflammation, interdiscipinarity, internal medicine, molecular analysis, oxidative stress

Interdisciplinary cooperation is particularly crucial in the fast-evolving fields of medicine and healthcare, which are progressively embracing scientific and technological breakthroughs. In this regard, there is a growing interconnection between medical specialties and other fields. More interdisciplinary care is needed, especially for challenging patients with numerous comorbidities, the majority of whom are usually elderly and fragile. One great challenge in health care arises at the intersection of numerous medical specializations. A multidisciplinary medical team is becoming more and more crucial due to the rapid advancement of medical knowledge. In order to improve patient care in the twenty-first century, multidisciplinary shared decision-making is necessary for the best diagnostic and treatment outcomes. Since each member of the healthcare team brings distinct expertise and skills, they all contribute to better case management. A well-functioning interdisciplinary team can reduce healthcare costs, enhance patient satisfaction, decrease medical errors and morbidity, and ultimately improve patient survival and overall quality of life.

The journal Frontiers in Immunology is presenting this Research Topic in recognition of the value of interdisciplinarity in the fields of medicine and research. In this Research Topic of *Frontiers in Immunology*, entitled “Interdisciplinarity in Internal Medicine from Basic Investigations to the Molecular Analysis,” we highlighted the latest findings regarding autoimmune disease, hemolytic uremic syndrome, COVID-19, septic shock, immune dysregulation in sepsis, and oxidative stress. This well-balanced SI consists of two original pieces and two case reports.

According to Fink et al., complement-mediated thrombotic microangiopathy (CM-TMA) is characterized by a unique set of factors, such as apparent late manifestation despite genetic predisposition, an insidious clinical course, COVID-19 as a potential triggering event, and a quick reaction to the long-acting C5 inhibitor ravulizumab. However, a previously unidentified disease course leading to end-stage kidney disease cannot be ruled out (1).

Sun et al. discovered a correlation between high SOFA scores and lipid compounds linked to age during sepsis, which may help explain why older patients had worse outcomes. A strong risk stratification model that incorporates six lipid biomarkers and patient age has been developed to accurately predict the risk of septic shock across age groups and in-hospital mortality, especially in older patients. The findings highlight the clinical value of plasma lipidomic analysis and offer novel therapeutic strategies that improve sepsis outcomes by focusing on host lipid metabolism. The stratified study emphasizes the importance of precise clinical diagnosis and treatment since various pathophysiological alterations may require customized treatments. Additionally, this study provides a strategy for identifying lipid biomarkers associated with immunosenescence in illnesses (2)..

The case report published by Gehrke et al. highlights the paradoxical immunological effects of TNF-α inhibition and the complex interplay between TNF-α and IL-17 signaling pathways. This led to a theory that explains the contradiction of TNF-blockade inducing autoimmunity in the setting of IL-17-mediated autoimmune illnesses and why anti-IL-17A/F therapy, rather than anti-TNF-α, should be utilized to treat VKHD (3).

In an observational pilot investigation, Padureanu et al. show a biologically coherent redox–inflammation–apoptosis coupling at presentation in acute pancreatitis. The authors found a modest, age-robust link between caspase-3 and both C-reactive protein (CRP) and protein carbonyls, as well as a strong intra-redox association between reduced glutathione (GSH) and protein carbonyls. The findings indicate a need for larger, adequately powered, multicenter prospective studies that evaluate parsimonious, pre-specified biomarker panels integrated with clinical and imaging-based scores and characterize the temporal dynamics of these markers using serial blood sampling (e.g., at 0, 24, 48, and 72 hours) (4).

We sincerely thank the authors who contributed papers to this Research Topic. The outstanding quality of these studies will surely open up novel opportunities for interdisciplinarity in internal medicine, from fundamental research to molecular analysis.
